# Increased Excursions to Functional Networks in Schizophrenia in the Absence of Task

**DOI:** 10.3389/fnins.2022.821179

**Published:** 2022-03-11

**Authors:** Miguel Farinha, Conceição Amado, Pedro Morgado, Joana Cabral

**Affiliations:** ^1^Life and Health Sciences Research Institute (ICVS), School of Medicine, University of Minho, Braga, Portugal; ^2^ICVS/3B's—PT Government Associate Laboratory, Braga/Guimarães, Portugal; ^3^Centro Clínico Académico Hospital de Braga, Braga, Portugal; ^4^Department of Mathematics and CEMAT, Instituto Superior Tècnico, University of Lisbon, Lisbon, Portugal; ^5^Center for Eudaimonia and Human Flourishing, Linacre College, University of Oxford, Oxford, United Kingdom; ^6^Center for Music in the Brain, Department of Clinical Medicine, Aarhus University, Aarhus, Denmark

**Keywords:** resting-state functional magnetic resonance imaging, dynamic functional connectivity, LEiDA, functional networks, dynamical systems theory, schizophrenia

## Abstract

Schizophrenia is a chronic psychotic disorder characterized by the disruption of thought processes, perception, cognition, and behaviors, for which there is still a lack of objective and quantitative biomarkers in brain activity. Using functional magnetic resonance imaging (fMRI) data from an open-source database, this study investigated differences between the dynamic exploration of resting-state networks in 71 schizophrenia patients and 74 healthy controls. Focusing on recurrent states of phase coherence in fMRI signals, brain activity was examined for intergroup differences through the lens of dynamical systems theory. Results showed reduced fractional occupancy and dwell time of a globally synchronized state in schizophrenia. Conversely, patients exhibited increased fractional occupancy, dwell time and limiting probability of being in states during which canonical functional networks—i.e., Limbic, Dorsal Attention and Somatomotor—synchronized in anti-phase with respect to the rest of the brain. In terms of state-to-state transitions, patients exhibited increased probability of switching to Limbic, Somatomotor and Visual networks, and reduced probability of remaining in states related to the Default Mode network, the Orbitofrontal network and the globally synchronized state. All results revealed medium to large effect sizes. Combined, these findings expose pronounced differences in the temporal expression of resting-state networks in schizophrenia patients, which may relate to the pathophysiology of this disorder. Overall, these results reinforce the utility of dynamical systems theory to extend current knowledge regarding disrupted brain dynamics in psychiatric disorders.

## 1. Introduction

Brain activity at “rest” captured using functional magnetic resonance imaging (fMRI) reveals the recurrent emergence and dissolution of connectivity patterns that overlap with functional networks typically activated during task (Beckmann et al., 2005; Fox and Raichle, 2007; Smith et al., 2009; Deco and Jirsa, 2012). These resting-state networks (RSNs) have been consistently detected and extensively analyzed across neuroimaging studies (Damoiseaux et al., 2006; van den Heuvel and Hulshoff Pol, 2010) and their characterization in the temporal domain—referred to as dynamic functional connectivity (dFC)—has been suggested to provide potential biomarkers of several neurological and psychiatric disorders (Sakoğlu et al., 2010; Hutchison et al., 2013; Preti et al., 2017). In fact, the discovery of biomarkers in dFC is crucial not only for more efficient diagnosis but also to inform computational models in order to gain insight into the large-scale organizational principles of brain activity in health and disease (Cabral et al., 2012a,b; Stefanovski et al., 2019; Courtiol et al., 2020; Kringelbach and Deco, 2020).

Schizophrenia (SZ) is a chronic brain disorder affecting 1 in 300 humans worldwide and, if left untreated, its symptoms can be persistent and disabling (James et al., 2018). The cognitive and behavioral symptoms observed in patients with SZ are hypothesized to arise from the disrupted functional integration of segregated brain areas (Friston et al., 1995; Liang et al., 2006; Lynall et al., 2010; Skudlarski et al., 2010). Furthermore, neuroimaging studies suggest that SZ patients have aberrant functional connectivity in brain networks and these abnormalities are related to disease symptoms (Wang et al., 2014). As such, neuroimaging data-sharing initiatives have been developed to potentiate the discovery of biomarkers of schizophrenia in fMRI signals, allowing to test novel analysis methods which may provide further insights into the pathophysiology of this disease and, potentially, lead to the discovery of new treatments for the diseased brain (Aine et al., 2017).

To date, previous studies investigating dFC have suggested that compared to healthy controls (HCs), patients with SZ spend more time in FC states characterized by weak connectivity (Rabany et al., 2019) and less time in FC states which represent strong, large-scale brain connectivity (Damaraju et al., 2014; Dong et al., 2018; Sanfratello et al., 2019). Furthermore, when SZ patients transition into the FC state of strongest connectivity, they switch states very rapidly (Rabany et al., 2019). Overall, SZ patients have been found to exhibit fewer changes between connectivity patterns compared to HCs (Miller et al., 2016). Notably, most research on dFC in SZ has been carried out using independent component analysis to extract time courses of networks which were subsequently used to estimate dFC through sliding-window analysis (SWA) (Sakoğlu et al., 2010; Preti et al., 2017). However, the choice of the window length affects the temporal resolution of the SWA approach—raising questions over its validity (Preti et al., 2017). In this study, to overcome this weakness, the Leading Eigenvector Dynamics Analysis (LEiDA) method, based on phase coherence of fMRI signals, is used to investigate dFC at an instantaneous level (Glerean et al., 2012; Cabral et al., 2017). It must be noted that methods based on phase coherence may fail to capture the non-linear stochastic nature of neuronal network dynamics to its full extent—prompting the use of metrics such as multi-scale-entropy (Courtiol et al., 2016). Nevertheless, methods based on phase coherence have demonstrated a particular sensitivity to alterations in psychiatric symptoms (both clinical and pre-clinical) motivating its use in the current work (Cabral et al., 2017; Figueroa et al., 2019; Lord et al., 2019; Alonso Mart-nez et al., 2020; Larabi et al., 2020).

The main aim in this study was to investigate if patients with SZ exhibit alterations in the dynamical exploration of functional networks during rest detected using LEiDA. Furthermore, this work examined the validity of the partitions resulting from the clustering procedure and investigated the influence of using the K-medoids algorithm instead of the *K*-means algorithm to differentiate SZ patients from HCs. This work hypothesized to find abnormal dFC in SZ patients characterized by reduced excursions to an FC state possibly involved in the integration of segregated functional connections and increased excursions to a number of FC states which represent functionally segregated networks.

## 2. Materials and Methods

### 2.1. Neuroimaging Data

Neuroimaging data was obtained from the publicly available repository COBRE preprocessed with NIAK 0.17—lightweight release (Calhoun et al., 2012; Bellec, 2016). The neuroimaging data included preprocessed resting-state fMRI (rs-fMRI) data from 72 SZ patients and 74 HCs, in which participants passively stared at a fixation cross (Aine et al., 2017). The rs-fMRI data featured 150 echo planar imaging volumes obtained in 5 min, with repetition time (TR) = 2 s, echo time = 29 ms, acquisition matrix = 64 × 64 mm^2^, flip angle = 75° and voxel size = 3 × 3 × 4 mm^3^. The acquisition and preprocessing of the fMRI data are fully described in detail in Bellec (2016). Preprocessing included slice-timing correction, coregistration to the Montreal Neurological Institute (MNI) template and resampling of the functional volumes in the MNI space at a 6 mm isotropic resolution. No confounds were regressed from the data, because this procedure may extract fMRI signal variance whose contribution to functional networks remains under debate (Murphy et al., 2013; Bright and Murphy, 2015; Nalci et al., 2019; Chen et al., 2020). In addition, the fMRI volumes were not spatially smoothed since the subsequent parcellation induces a level of smoothing. Furthermore, temporal filters were not applied since previous works considering the whole-frequency spectrum have been shown to improve within-subject reliability regarding the temporal expression of FC patterns (Vohryzek et al., 2020).

Inspection of the fMRI data for each subject resulted in the exclusion of one subject whose data did not include all 150 volumes. Therefore, the final dataset used in this analysis included 71 SZ patients (57 males) and 74 HCs (51 males). A goodness of fit χ^2^ test did not reject the null hypothesis of independence between gender and group (*p* = 0.1167). Both groups had an age range of 18–65 years old. A two-sided Wilcoxon Rank-Sum test with Bonferroni correction did not identify a significant difference between the mean age of the groups (*p* = 0.4253). The framewise displacement (FD) provided a quantitative indication of each subject's head motion during the scanning period (Power et al., 2012). The same statistical test detected a significant intergroup difference in the group mean FD (*p* < 0.001). Specifically, on average, the fMRI signals of SZ patients were characterized by larger amounts of head motion (FD). Given the statistically significant difference in the mean FD, the impact of head-motion in the group-level results was investigated (see Supplementary Material Section 2).

### 2.2. Parcellation

The entire brain of each participant was parcellated into 90 cortical and sub-cortical non-cerebellar regions using the Anatomic Automatic Labeling (AAL) template. Accordingly, for each region in the brain template, the fMRI signals were averaged over all voxels belonging to that brain area. For each subject, this resulted in an *N*× *T* dataset, where *N* = 90 is the number of brain areas and *T* = 150 is the number of volumes in each scan.

### 2.3. Computation of Dynamic Functional Connectivity

**Figure 1:**
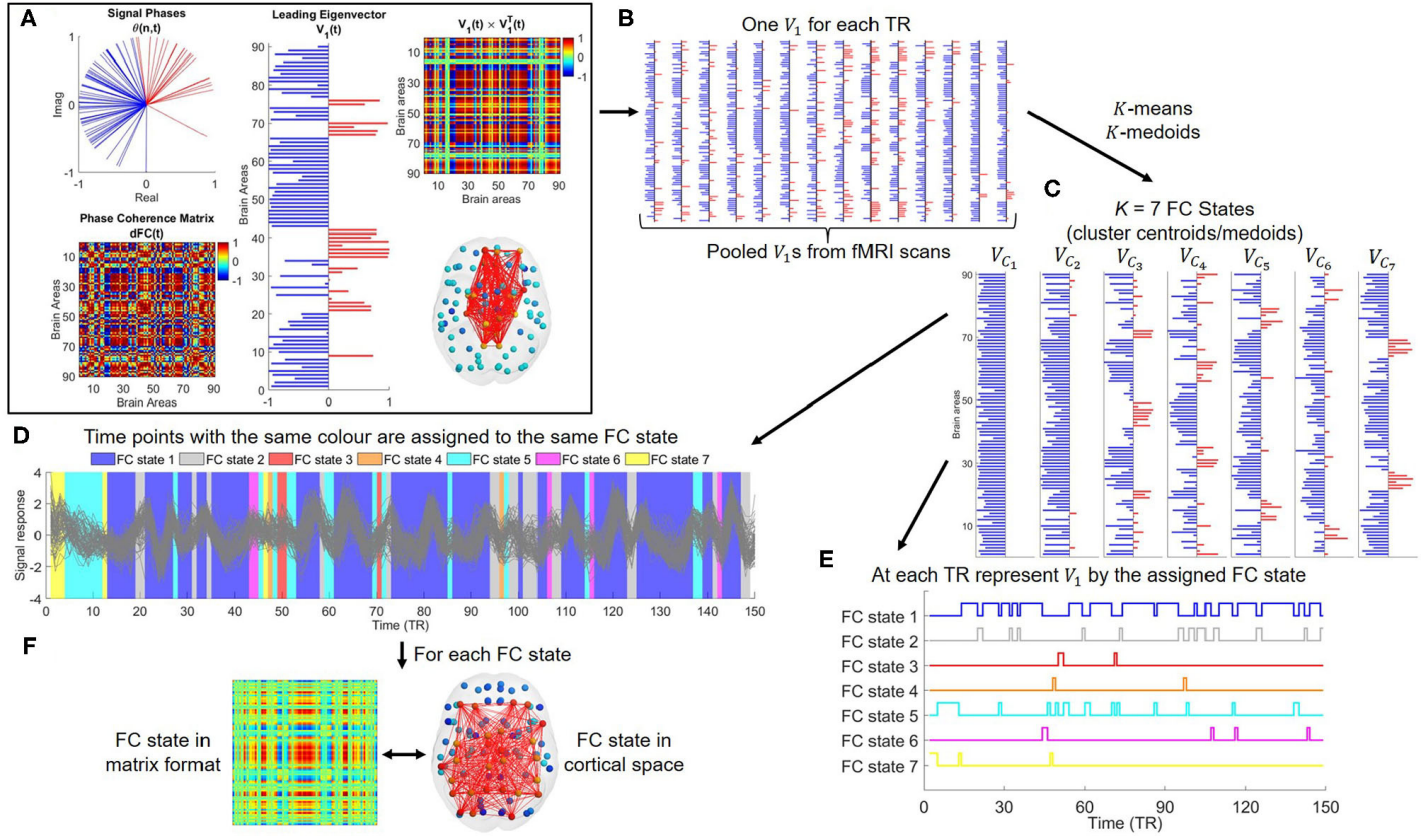
Graphical illustration of the estimation and characterization of the temporal trajectories of recurrent FC states obtained by using Leading Eigenvector Dynamics Analysis (LEiDA). **(A)** Phases of all *N* = 90 brain areas in the complex plane at time *t* (top left); Phase coherence matrix at time *t*, *dFC*(*t*) (bottom left); Vector representation of the leading eigenvector, *V*_1_(*t*), of *dFC*(*t*) (middle); Matrix representation of *V*_1_(*t*) (top right); Network representation of *V*_1_(*t*), with links between the areas with positive elements in *V*_1_(*t*) plotted in red (bottom right). **(B)** The leading eigenvectors are computed for each time point and from all fMRI scans. **(C)** The pooled leading eigenvectors are partitioned into *K* clusters using a clustering algorithm. The cluster centroids/medoids are assumed to represent recurrent patterns of phase coherence (FC states). **(D,E)** The leading eigenvector at each TR is represented by the centroid/medoid of the cluster to which it was assigned by the clustering procedure. This originates time courses of FC states for each fMRI session. The time courses are then characterized using tools from dynamical systems theory. **(F)** Each FC state can be represented as a *N* × *N* matrix (outer product) and as a network in cortical space (elements with positive sign linked by red edges).

To compute the phase relationship between each pair of AAL regions, first the instantaneous phase of the fMRI signals across all brain regions *n*∈{1, …, *N*} for each time *t*∈{2, …, *T*−1}, θ(*n, t*), were estimated by computing the Hilbert transform of their regional time courses (Glerean et al., 2012). Here, the first and last TR of each fMRI scan were excluded due to possible signal distortions induced by the Hilbert transform (Vohryzek et al., 2020). The Hilbert transform enables the capture of the time-varying phase of a fMRI signal at each time, *t*, by converting it into its analytical representation [see Figure 1A (top left)] (Glerean et al., 2012; Cabral et al., 2017). To obtain a whole-brain pattern of phase synchrony, the phase coherence between areas *n* and *p* at each time *t*, *dFC*(*n, p, t*), was estimated using Equation (1):


(1) $$\begin{array}{l} dFC\left(n,p,t\right)=cos\left(\theta \left(n,t\right)-\theta \left(p,t\right)\right) \end{array}$$

where phase coherence values range between -1 (areas *n* and *p* in anti-phase at time *t*) and 1 (areas *n* and *p* have synchronized signals at time *t*), as shown in Figure 1A (bottom left). This computation was repeated for all pairwise combinations of brain areas (*n, p*), with *n, p*∈{1, …, 90}, at each time point *t*, with *t*∈{2, …, 149}, and for all subjects. For each subject, the resulting dFC was a three-dimensional tensor with dimension *N* × *N* × *T*′, where *T*′ = 148, i.e., 148 *dFC*_90 × 90_(*t*) matrices were estimated.

### 2.4. Functional Connectivity Leading Eigenvector

To characterize the evolution of the phase coherence matrix over time with reduced dimensionality, the current study employed the LEiDA method which considers only the leading eigenvector, *V*_1_(*t*), of each *dFC*(*t*) matrix (Cabral et al., 2017). In detail, as observed in Figure 1A (middle), the leading eigenvector, *V*_1_(*t*), is an *N* × 1 vector that captures the dominant connectivity pattern of phase coherence at time *t*, i.e., *V*_1_(*t*) represents the main orientation of the phases over all brain areas (Cabral et al., 2017). Under this framework, for each time *t*, the associated leading eigenvector partitions the *N* brain areas into two communities by separating the elements with different signs in *V*_1_(*t*) (Newman, 2006; Cabral et al., 2017). When all elements of *V*_1_(*t*) have the same sign, the phases between brain regions are coherent, which is indicative of a global mode of phase coherence governing all fMRI signals. This implies that all brain regions belong to the same community. Contrarily, if the elements of *V*_1_(*t*) have different signs (i.e., positive and negative), the connectivity pattern between brain regions is not coherent. As a result, each brain area is assigned to one of the two communities according to their phase relationship. Additionally, the absolute value of each element in the leading eigenvector weighs the contribution of each brain area to the assigned community (Newman, 2006; Cabral et al., 2017). The dominant FC pattern of the *dFC* matrix at time *t* can also be reconstructed back into matrix format by computing the (*N* × *N*) outer product $V_{1}\left(t\right)V_{1}^{⊺}\left(t\right)$, as shown in Figure 1A (top right). Given that if *V*_1_(*t*) is a leading eigenvector, so is −*V*_1_(*t*), following the procedure of Figueroa et al. (2019); Lord et al. (2019); Vohryzek et al. (2020), it was ensured that most of the elements in *V*_1_(*t*) had negative values. This is because by assigning positive values to the brain areas whose phases did not follow the global mode, functional brain networks were distinctly detected, as seen in Figure 1A (bottom right). Importantly, this approach was found to explain most of the variance of observed phase coherence data variation, while substantially reducing its dimensionality. In fact, the leading eigenvector accounted for more than 50% of the variance in phase coherence at all time points and for all subjects.

### 2.5. Estimation of FC States

Upon computing the leading eigenvector of the phase coherence matrix for each recording frame, the next step in the analysis was to characterize the evolution of the dFC over time by identifying recurrent FC states in the data, as illustrated in Figure 1C (Cabral et al., 2017).

The dataset of all leading eigenvectors computed across all 145 participants at the set of volumes {2, …, 149}, totalling 148 × 145 = 21, 460 leading eigenvectors, was clustered using: (1) the *K*-means algorithm; and (2) the *K*-medoids algorithm (Aggarwal and Reddy, 2013). Here, both algorithms were run with a value of *K* from 2 to 20, i.e., dividing the set of leading eigenvectors into *K* = {2, 3, …, 20} clusters. Furthermore, in both clustering analyses, the cosine distance was used as the distance metric for minimization and the algorithms were run 1,000 times to minimize the chances of getting trapped in a local minima (Cabral et al., 2017; Figueroa et al., 2019; Lord et al., 2019; Vohryzek et al., 2020).

Independently of the algorithm, the LEiDA clustering procedure outputs one optimal clustering solution for each value of *K* clusters. Specifically, each clustering solution contains *K* clusters *C* = {*C*_1_, …, *C*_*K*_}, with *K*∈{2, …, 20}—decomposing the *N*-dimensional phase space of pooled leading eigenvectors into a *K*-dimensional state space. Each cluster *C*_α_ (α∈{1, …, *K*}) is represented by a vector of dimension *N* × 1, *V*_*C*_α__, which represents a recurrent FC state, as depicted in Figure 1C. It must be noted that the *K*-means and *K*-medoids algorithms provide distinct interpretations regarding the functional meaning of the mentioned FC states. According to the *K*-means algorithm, the prototypes of each cluster, designated as centroids, are given by the mean of the leading eigenvectors belonging to each cluster. As such, centroids may not correspond to actual data points from the set of leading eigenvectors. On the other hand, the *K*-medoids algorithm chooses actual leading eigenvectors as the prototypes of the clusters which are designated as medoids. Whilst at the cost of higher computational complexity, the robustness of the *K*-medoids algorithm means it is better suited to manage outliers than the *K*-means algorithm when detecting recurrent FC states (Aggarwal and Reddy, 2013). Assuming that some of the leading eigenvectors belonging to SZ patients were outliers, by employing the *K*-medoids algorithm, their influence would be underestimated when detecting FC states—resulting in more representative functional networks of the set of leading eigenvectors from both groups.

### 2.6. Characterization of FC State Trajectories

For each clustering solution, the set of estimated *K* FC states was used to obtain, for each participant, time courses of FC states (as represented in Figures 1D,E). This was accomplished by representing each *V*_1_ at time *t* by the FC state (centroid/medoid) of the cluster to which it was assigned by the clustering algorithm, depicted as a matrix and as a network in cortical space in Figure 1F. Specifically, following the conceptual framework proposed by Vohryzek et al. (2020), resting-state fMRI time series were assumed to temporally evolve through a finite state trajectory of recurrent patterns of phase coherence. Following this rationale, each clustering solution with *K* FC states was assumed to define a finite state space *S* = {1, …, *K*}. Furthermore, for a clustering solution with *K* clusters, the cluster (FC state) to which *V*_1_ was assigned at time *t*, denoted by *V*_*t*_, was assumed to define a stochastic process, {*V*_*t*_:*t*∈{2, …, 149}}, with an associated finite state space given by *S*. Consequently, considering the Markov property (Kulkarni, 2011) holds, each temporal trajectory of FC states was assumed to define a time-homogeneous Discrete Time Markov Chain (DTMC). Importantly, it must be noted that, although brain activity is an uninterrupted process, the restricted fMRI scanning windows implied the state trajectories were temporally limited—resulting in a number of DTMCs not spanning the entire state space.

A number of descriptive measures were considered to characterize the properties of the temporal trajectories of FC states observed in SZ patients and HCs. Notably, these measures have been shown to provide relevant insights on dynamic brain activity in previous LEiDA analyses (Cabral et al., 2017; Figueroa et al., 2019; Lord et al., 2019).

#### 2.6.1. Fractional Occupancy

The fractional occupancy (probability of occurrence) of an FC state α represents the proportion of times *V*_*t*_ is assigned to cluster *C*_α_ throughout a scan (Vohryzek et al., 2020). The fractional occupancy of FC state α for the fMRI scan of subject *s*, $P_{\alpha }^{\left(s\right)}$, is calculated (estimation) as follows:


(2) $$\begin{array}{l} P_{\alpha }^{\left(s\right)}=\frac{1}{T^{\prime }}\sum_{t=1}^{T^{\prime }}1_{\left\{V_{t}^{\left(s\right)}\in C_{\alpha }\right\}}\;,\;\alpha \in \{1,\ldots ,K\} \end{array}$$

where *T*′ = 148 is the number of time points (first and last volume of each scan were excluded), 1 is the indicator function and $V_{t}^{\left(s\right)}$ is the FC state to which *V*_1_(*t*) was assigned at time *t*. For each clustering solution, this measure was estimated for each of the *K* FC states separately for each fMRI scan.

#### 2.6.2. Dwell Time

The dwell time (mean duration) of an FC state represents the mean number of consecutive epochs spent in that state throughout the duration of a scan (Vohryzek et al., 2020). The dwell time of FC state α, $DT_{\alpha }^{\left(s\right)}$, is defined (estimation) as:


(3) $$\begin{array}{l} DT_{\alpha }^{\left(s\right)}=\frac{1}{k_{\alpha }^{\left(s\right)}}\overset{k_{\alpha }^{\left(s\right)}}{\underset{d_{\alpha }=1}{\sum }}R_{d_{\alpha }},\alpha \in \left\{1,\ldots ,K\right\} \end{array}$$

where $k_{\alpha }^{\left(s\right)}$ is the number of consecutive periods in which $V_{t}^{\left(s\right)}$ was assigned to cluster *C*_α_ and *R*_*d*_α__ is the duration of each of the $k_{\alpha }^{\left(s\right)}$ periods. For each clustering solution, the dwell time was estimated for each of the *K* FC states separately for each fMRI scan.

#### 2.6.3. One-Step Transition Probability Matrix

Considering a clustering solution with state space *S* = {1, …, *K*}, the probability of being in FC state α at time *t* and transition to FC state β at time *t*+1 is given by the following expression:


(4) $$\begin{array}{l} P_{\alpha \beta }^{\left(s\right)}=\frac{1}{T^{\prime }-1}\sum_{t=1}^{T^{\prime }-1}1_{\left\{V_{t}^{\left(s\right)}\in \;C_{\alpha }\;,\;V_{t+1}^{\left(s\right)}\in \;C_{\beta }\right\}} \end{array}$$

with α, β∈{1, …, *K*} (Vohryzek et al., 2020). From Equation (4), for a clustering solution with *K* FC states, it follows that the Transition Probability Matrix (TPM) of the fMRI scan of subject *s*, **P**^(*s*)^, is defined (estimation) as:


(5) $$\begin{array}{l} P^{\left(s\right)}=P[V_{t+1}^{\left(s\right)}\in C_{\beta }\;|\;V_{t}^{\left(s\right)}\in C_{\alpha }]=\frac{P_{\alpha \beta }^{\left(s\right)}}{P_{\alpha }^{\left(s\right)}} \end{array}$$

with α, β∈{1, …, *K*}. For the tentative optimal clustering solution, a TPM was estimated separately for the DTMC of each fMRI scan.

#### 2.6.4. Limiting Probability

In this study, the limiting distribution was only estimated for irreducible and aperiodic DTMCs (Kulkarni, 2011), with finite state space given by the tentative optimal state trajectories. Therefore, for every subject, *s*, with a DTMC satisfying the aforementioned criteria, it followed that:


(6) $$\begin{array}{l} \underset{t\to \infty }{\lim}{\left(P_{\alpha \beta }^{\left(s\right)}\right)}^{t}=\pi_{\beta }^{\left(s\right)}>0,\alpha ,\beta \in \left\{1,\ldots ,K\right\} \end{array}$$

where the estimate of the row vector denoting the stationary distribution of the DTMC (Kulkarni, 2011), $\pi^{\left(s\right)}=[\pi_{\beta }^{\left(s\right)}]{\;}_{\beta \in S}$, with dimension 1 × |*S*|, is given by:


(7) $$\begin{array}{l} \pi^{\left(s\right)}=1\times {\left(\text{I}-{\text{P}}^{\left(s\right)}+\text{ONE}\right)}^{-1} \end{array}$$

where **1** is a 1 × |*S*| vector of ones, **I** is the identity matrix with rank |*S*|, **P**^(*s*)^ is the TPM of subject *s* and **ONE** is an |*S*| × |*S*| matrix all of whose entries are one. Due to the inclusion criteria imposed on the DTMCs defined by the optimal state trajectories, i.e., irreducibility and aperiodicity, only 37 and 46 DTMCs from the HC and SZ groups, respectively, were analyzed. For a given FC state β, π_β_ (element β of the row vector ***π***) was the measure to be used to perform intergroup comparisons. Importantly, since only aperiodic DTMCs were considered, π_β_ can be understood as the limiting probability that the DTMC is in FC state β and as the long-run fraction of time the DTMC spends in FC state β. It must be noted that intergroup comparisons between the estimated stationary distributions were not performed in this study.

### 2.7. Intergroup Comparisons

In this research, hypothesis tests to compare the group mean of the properties calculated from the temporal state trajectories observed in SZ patients and HCs were performed using Monte Carlo permutation tests (Pesarin and Salmaso, 2010) by adapting the procedure used by Cabral et al. (2017); Figueroa et al. (2019); Lord et al. (2019). To produce an accurate approximate estimation of the permutation distribution, these tests were conducted using *B* = 10, 000 permutations (Marozzi, 2004). Here, depending on the result of a Levene's test (Levene, 1960) (used to assess the homogeneity between the group variances) the Monte Carlo permutation tests were performed based on one of the following two statistics (under the null hypothesis):


(8) $$\begin{array}{l} T_{0}^{*}=\{\begin{array}{ll} \frac{\bar{X_{1}}-\bar{X_{2}}}{\sqrt{\frac{S_{1}^{2}}{n_{\text{HC}}}+\frac{S_{2}^{2}}{n_{\text{SZ}}}}}, & p_{\text{Levene's test}}<0.05\\ \frac{\bar{X_{1}}-\bar{X_{2}}}{\sqrt{S_{\text{p}}(\frac{1}{n_{\text{HC}}}+\frac{1}{n_{\text{SZ}}})}}, & \text{otherwise} \end{array} \end{array}$$

where $\bar{X_{1}}$ and $\bar{X_{2}}$ are the random sample means, *S*_1_ and *S*_2_ are the random sample standard deviations, and *n*_HC_ and *n*_SZ_ are the sample sizes for the HC and SZ groups, respectively. The pooled random standard deviation, *S*_p_, is given by $S_{\text{p}}=\left((n_{\text{HC}}-1\right)S_{1}^{2}+\left(n_{\text{SZ}}-1\right)S_{2}^{2})/(n_{\text{HC}}+n_{\text{SZ}}-2)$. Under the null hypothesis, the statistic from Equation (8) used to perform the statistical test was subsequently used to obtain the value of the statistic under each of the *B* permutations of the sample data. In this study, the standard deviation of the difference of the group means was estimated using 500 bootstrap samples within each permutation sample. This was performed so that the estimation of this quantity was conducted independently of the calculated means difference.

To complement the statistical hypothesis tests and understand the magnitude of the detected intergroup differences independently of the sample size, the effect size was estimated using Hedge's g statistic (Hedges, 1981). The use of this measure was based on its appropriateness to measure the effect size for the difference between means and on the fact that this measure takes into account the size of the sample from each group.

### 2.8. Comparison to Resting-State Functional Networks

The functional relevance of the estimated FC states was investigated by assessing whether there was a significant spatial overlap between the centroids/medoids and any of the seven reference RSNs defined by Thomas Yeo et al. (2011). This was accomplished by employing the procedure used by Lord et al. (2019); Vohryzek et al. (2020). Specifically, the seven RSNs were transformed into seven non-overlapping vectors with dimension 1 × 90, where each entry of the vectors corresponded to the proportion of voxels of each AAL brain area that were assigned to each of the seven RSNs. Finally, the Pearson correlation coefficient was used to assess the spatial overlap between these seven RSNs and the centroids/medoids *V*_*C*_α__ with α∈{1, …, *K*} (all negative values of *V*_*C*_α__ were set to zero so that only areas thought to define relevant functional networks were considered).

### 2.9. Unsupervised Internal Cluster Validation Criteria

The quality of clustering solutions outputted by the clustering algorithms was evaluated using the average Silhouette coefficient and the Dunn's index (Aggarwal and Reddy, 2013).

### 2.10. External Validation Clustering Agreement Measures

Clustering outputs from distinct algorithms were compared using the Adjusted Rand Index (ARI) and the Variation of Information (VI) clustering agreement measures (Aggarwal and Reddy, 2013).

### 2.11. Clustering Stability Evaluated by K-Fold Cross-Validation

The stability of clustering solutions was assessed according to a 10-fold cross-validation procedure adapted from Martins and Cardoso (2008). Firstly, the sample of the pooled leading eigenvectors was split into two subsamples, referred to as training and test samples. Secondly, a clustering algorithm was applied to the training sample—yielding partition *P*_1_. Subsequently, a Nearest Centroid classifier assigned each observation of the test sample to the cluster of partition *P*_1_, whose centroid was nearest—resulting in the class set *P*_2_ of the test sample. The same clustering algorithm was then applied to the test sample—producing the cluster set *P*_3_. Finally, partitions *P*_2_ and *P*_3_ were compared based on the ARI, VI and percent agreement (fraction of objects correctly assigned). This procedure was repeated for each of the 10 cross-validation folds.

### 2.12. Software

This analysis used MATLAB R2019b (MATLAB, 2019), the Statistics and Machine Learning Toolbox^TM^ and the Econometrics Toolbox^TM^.

## 3. Results

### 3.1. Intergroup Differences Across Partition Models Detected by the K-Means Algorithm

The collection of clustering solutions was investigated to search for FC states whose fractional occupancy and dwell time most significantly and consistently differed between SZ patients and HCs. For a partition model with *K* clusters, *K* hypothesis tests were performed. Consequently, to account for the increased probability of false positives, the significance threshold α_1_ = 0.05 was adjusted to α_2_ = 0.05/*K* using a Bonferroni correction. Additionally, a conservative significance threshold of $\alpha_{3}=0.05/\overset{20}{\underset{K=2}{\sum }}K$ was considered to encompass both dependent and independent null hypotheses across clustering solutions.

**Figure 2:**
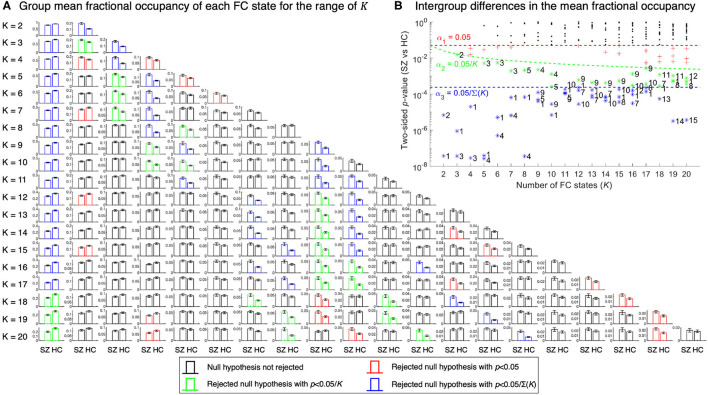
Intergroup comparisons of the mean fractional occupancy of each FC state for each clustering solution. **(A)** Barplot of the estimated mean fractional occupancy with associated standard error of each FC state for each group. For each FC state, the color of the bars indicates whether the null hypothesis of no intergroup differences in the mean fractional occupancy was rejected (two-tailed tests). The standard error of each bar was calculated as the standard deviation of the sample data divided by the square root of the sample size. **(B)** Two-sided *p*-values obtained for the intergroup comparisons of the mean fractional occupancy of each FC state for each partition model. FC states (clusters) are ranked according to their probability of occurrence, where cluster 1 consists of the largest number of objects and cluster *K* consists of the least number of objects. The red, green and blue dotted lines correspond to a 0.05, 0.05/*K* and $0.05/\overset{20}{\underset{K=2}{\sum }}K$ significance threshold, respectively.

Figure 2B presents, for each clustering solution, the *K* two-sided *p*-values obtained from evaluating whether the group mean fractional occupancy of an FC state differed between SZ patients and HCs. From the inspection of Figure 2B, it is apparent that, across all partition models, the clustering procedure consistently returned FC states whose mean fractional occupancy differs significantly between groups—falling below the corrected significance thresholds α_2_ and α_3_.

Closer inspection of Figure 2B shows there are significant intergroup differences in the mean fractional occupancy of FC state 1 for a range of clustering solutions (*p* < α_3_, two-tailed tests). In fact, the mean fractional occupancy of this state was found to be significantly decreased in SZ patients compared to HCs (*p* < α_3_ for *K*∈{2, …, 17}, one-tailed tests), as suggested in Figure 2A. Interestingly, for all partition models, the centroid associated with FC state 1 revealed this recurrent FC pattern represents a globally synchronized state of phase coherence (all elements of the centroid had the same sign). Hence, FC state 1 is referred to as the Global Mode.

As depicted in Figure 2B, across all clustering solutions, further non-global FC states are characterized by significant intergroup differences in the group mean fractional occupancy (*p* < α_3_, two-tailed tests). Interestingly, all these states were typified by a higher mean probability of occurrence in the SZ group compared to the HC group (*p* < α_3_, one-tailed tests), as presented in Figure 2A. Visual inspection of these non-global FC states revealed they represent varying forms of the same underlying connectivity patterns. Specifically, states detected for lower values of *K* could be obtained by combining the fine-grained FC patterns identified in partition models with larger values of *K*—evidencing the dependence among the hypothesis tests performed across clustering solutions.

The analysis of mean dwell time estimates of detected FC states suggested that this measure did not allow as much consistent and clear differentiation between groups compared to the estimates of the fractional occupancy of FC states, as observed in Supplementary Figure 1. In fact, the mean dwell time of the Global Mode was reduced significantly in SZ patients compared to HCs in only 8 clustering solutions (*p* < α_3_, one-tailed tests). Conversely, across all partition models, the mean dwell time was identified as significantly increased in the SZ group compared to the HC group in only two FC states (*p* < α_3_, one-tailed tests). Notably, these non-global states were highly correlated (Pearson's *r* = 0.996)—reinforcing the fact that significant intergroup differences were consistently detected across similar FC patterns.

### 3.2. Overlap With Reference Functional Networks

Investigation of the overlap between the centroids of the detected FC states and the seven canonical functional networks defined by Thomas Yeo et al. (2011), depicted in Supplementary Figure 2B, confirmed that intergroup differences were consistently detected in a number of varying forms of the same FC patterns. Interestingly, FC state 1 did not significantly overlap with any of the seven reference RSNs, as shown in Supplementary Figure 2A—indicating this global state does not reveal the activation of any particular subset of functionally coupled brain regions. Additionally, across partition models, the non-global FC states with a significantly increased mean fractional occupancy in SZ were found to repeatedly overlap with the Somatomotor, Dorsal Attention and Limbic networks, as illustrated in Supplementary Figure 2A. The mean dwell time of FC states related to the Dorsal Attention network was significantly increased in the SZ group compared to the HC group. Accordingly, FC states with functional activity possibly related to that of the aforementioned canonical RSNs recur more often (and lasted for larger consecutive periods of time) in SZ patients.

### 3.3. Internal Validation of K-Means Clustering Solutions

**Figure 3:**
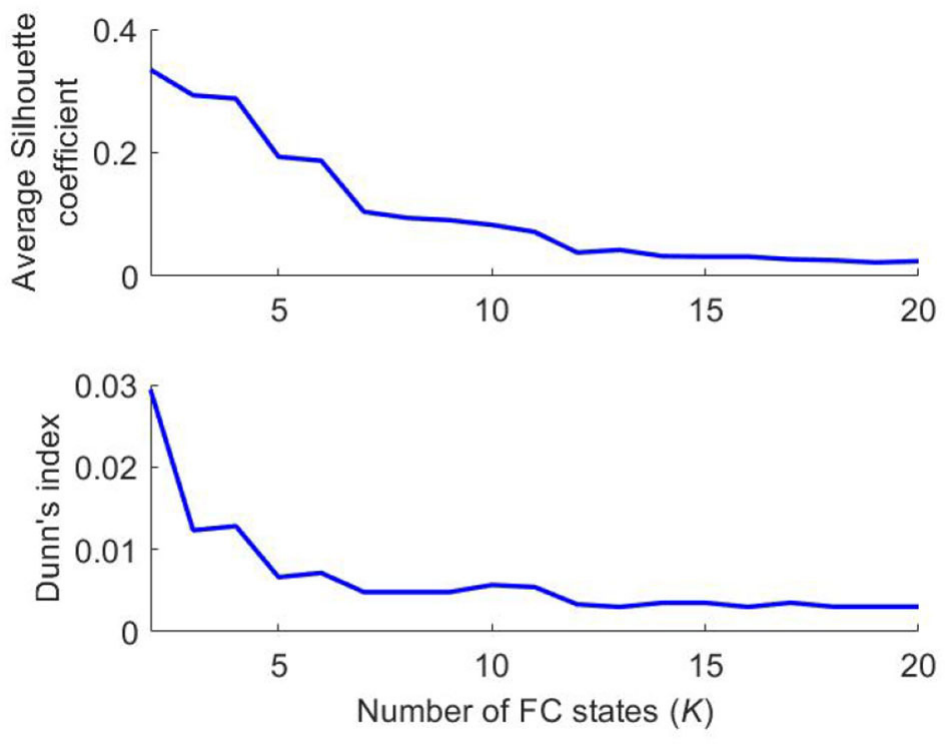
Internal validation of *K*-means clustering results. Average Silhouette coefficient and Dunn's index used to evaluate the quality of clustering solutions.

As shown in Figure 3, the highest average Silhouette coefficient and Dunn's index were obtained for clustering solutions with a low number of FC states, which are of limited interest for the present study. Contrarily, for clustering solutions with more than 12 clusters, both validation measures remained relatively constant at low values—indicating such partitions are also of limited interest.

Notably, for clustering solutions with *K* between 7 and 11, the average Silhouette coefficient decreased smoothly and the Dunn's index remained approximately constant, as observed in Figure 3—suggesting these partition models are of potential interest for further analysis.

### 3.4. Selection of the Optimal Clustering Solution

For the subsequent analysis, the partition model with 11 FC states was selected as the optimal *K*-means clustering solution. This decision was based upon the ability to identify a collection of FC states with properties that significantly differed between groups and the quality and stability of the actual partition of the data.

**Figure 4:**
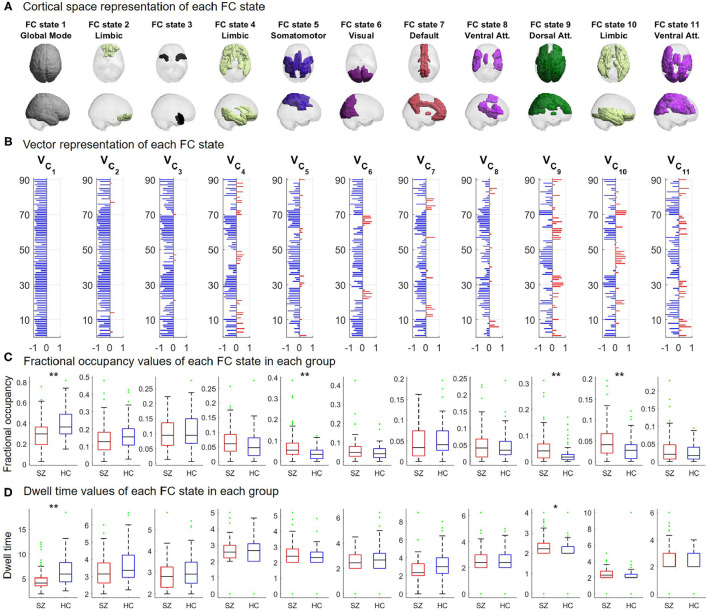
Repertoire of FC states defined from phase coherence obtained from the clustering solution with *K* = 11 clusters. The FC states are arranged (left-to-right) according to decreasing estimated fractional occupancy. Each FC state is represented by a *N* × 1 centroid *V*_*C*_α__, with α∈{1, …, 11}. **(A)** Cortical rendering of all brain areas with positive values in *V*_*C*_α__. The functional network defined by Thomas Yeo et al. (2011) with which *V*_*C*_α__ most significantly overlapped is indicated as subtitle. **(B)** Vector representation showing the *N* elements in *V*_*C*_α__, representing the contribution of each brain area to FC state α. **(C)** Boxplot of the fractional occupancy values for each FC state for the SZ and HC groups. **(D)** Boxplot of the dwell time values for each FC state for the SZ and HC groups. Single and double asterisks indicate significant intergroup differences with *p* < α_2_ and *p* < α_3_ (one-tailed tests), respectively. Green points represent outliers, according to the Tukey criterion (Tukey, 1977).

The collection of 11 phase coherence patterns, their fractional occupancy and dwell time values are presented in Figure 4. The non-global FC states were found to be significantly correlated with six of the seven RSNs estimated by Thomas Yeo et al. (2011), as shown in Figure 4A. From Figure 4B, it is apparent that the 11 FC states represent phase coherence between distinct subsets of brain areas. Furthermore, significant intergroup differences were identified in the mean fractional occupancy of 4 FC states, as observed in Figure 4C. Closer inspection of Figures 4A,B reveals that these 4 FC states represent distinct functionally meaningful networks. The mean fractional occupancy of FC state 1 was significantly decreased in SZ patients compared to HCs (*p* < α_3_; Hedge's *g* = 0.694, medium to large effect size), with estimates 29.8 ± 19.8% and 39.8 ± 14.9% (mean ± std) for the SZ and HC groups, respectively. Furthermore, the mean fractional occupancy of FC states 5, 9, and 10 was significantly increased in SZ patients compared to HCs (*p* < α_3_; Hedge's *g* = {0.611, 0.630, 0.629}, respectively, medium to large effect size), with values 7.61 ± 7.78%, 5.67 ± 6.34%, and 5.14 ± 4.09% for the SZ group and 4.02 ± 3.15%, 2.52 ± 3.24%, and 2.94 ± 2.81% for the HC group, respectively (mean ± std). Finally, the mean dwell time of 2 FC states was found to be significantly different between groups, as shown in Figure 4D. Specifically, the mean dwell time of the Global Mode was significantly decreased in SZ patients compared to HCs (*p* < α_3_; Hedge's *g* = 0.618, medium to large effect size). The mean dwell time value of this state was 4.986 ± 2.306 s and 6.574 ± 2.803 s (mean ± std) for SZ patients and HCs, respectively. The mean dwell time of FC state 9 was significantly increased in SZ patients compared to HCs (*p* < α_2_; Hedge's *g* = 0.519, medium to large effect size), with values 2.287 ± 0.763 s for the SZ group and 1.830 ± 0.977 s for the control group (mean ± std).

Furthermore, the percent agreement, ARI and VI obtained for each fold of the 10-fold cross-validation procedure are provided in Supplementary Table 1. The results suggest respectively, good levels of association and paired agreement between partitions of the test sample and that the amount of information that was lost in changing from the class set *P*_2_ to the cluster set *P*_3_ of the test sample was relatively low. Consequently, the optimal clustering solution is considered valid and appropriate for further analysis.

### 3.5. State-to-State Transitions of the Optimal State Trajectories

**Figure 5:**
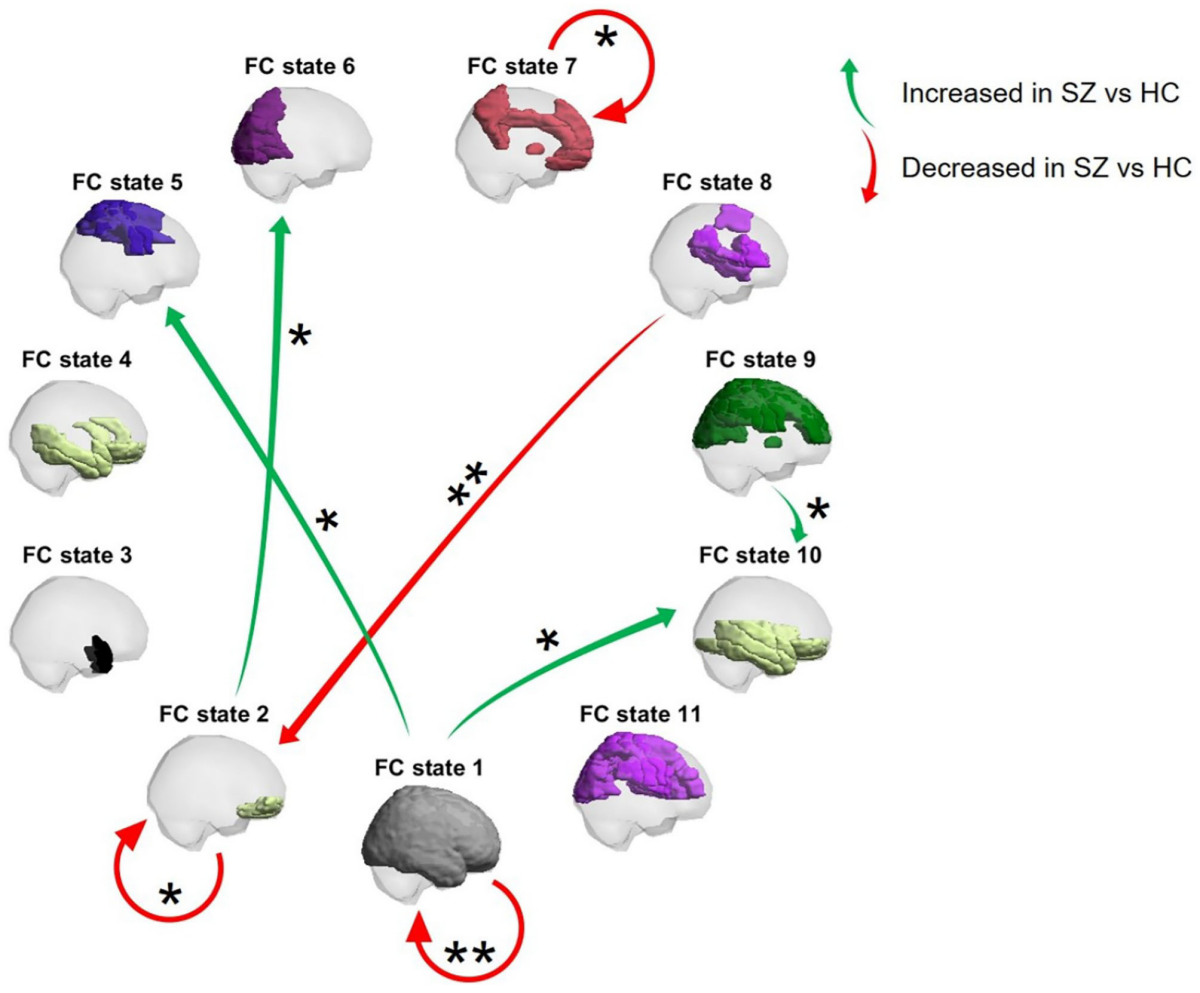
Transition diagram of the state-to-state transitions significantly altered in SZ patients compared to HCs. Arrows represent a mean transition probability that was significantly increased (green) or decreased (red) in SZ patients compared to HCs. Single and double asterisks indicate, respectively, significant intergroup differences with *p* < 0.05/11 and *p* <0.05/(11 × 11) (one-tailed tests).

With respect to the optimal clustering solution, for all participants, the individual DTMC defined by the temporal trajectories through the finite state space *S*′ = {1, …, 11}, was characterized by its estimated TPM. For each probability of transitioning from state α to state β (α → β, α, β∈*S*′), a two-sided *p*-value was obtained by testing whether its group mean differed between groups. The state-to-state transition probabilities that were significantly affected in SZ patients compared to HCs are depicted in Figure 5.

As shown in Figure 5, the mean probability of remaining in FC state 1 was significantly reduced in SZ patients compared to HCs (Hedge's *g* = 0.726, medium to large effect size). Furthermore, the mean probability of remaining in FC states 2 and 7 was significantly reduced in the SZ group compared to the HC group (Hedge's *g* = {0.449, 0.515}, respectively, small to medium effect size). Lastly, the mean probability of transitioning from FC state 1 to FC states 5 and 10 (Hedge's *g* = {0.452, 0.513}, respectively, small to medium effect size) and from FC state 9 to FC state 10 (Hedge's *g* = 0.461, small to medium effect size) were significantly increased in SZ patients compared to HCs. Overall, a number of mean transition probabilities were found to be altered in SZ patients.

### 3.6. Limiting Probabilities of the Optimal FC States

For the subgroup of 37 HCs and 46 SZ patients with irreducible and aperiodic DTMCs, the estimated mean long-run proportion of TRs spent in FC state 1 was, respectively, 0.309 ± 0.104 and 0.272 ± 0.111 (mean ± std). Surprisingly, no intergroup differences were found in the mean limiting probability of this state (two-tailed test; Hedge's *g* = 0.342, small to medium effect size). Only the mean limiting probability of FC states 5 and 10 were identified as significantly increased in the SZ subgroup compared to the HC subgroup (*p* < 0.05, one-tailed tests; Hedge's *g* = {0.464, 0.449}, respectively, small to medium effect size).

### 3.7. Influence of Using the K-Medoids Algorithm Instead of the K-Means Algorithm

The application of the *K*-medoids algorithm was found to enable the detection of FC states with a mean fractional occupancy and a mean dwell time that consistently and significantly differ between groups. Similarly with the findings produced by the *K*-means algorithm, the *K*-medoids algorithm identified an FC state which represents a globally synchronized state whose mean fractional occupancy and dwell time was significantly decreased in SZ patients compared to HCs. Additionally, the mean fractional occupancy of a number of non-global FC states related to the reference Somatomotor, Dorsal Attention and Limbic RSNs was found to be significantly increased in SZ patients compared to HCs. Finally, the mean dwell time of FC states related to the Dorsal Attention and Limbic networks was found to be significantly increased in SZ patients compared to HCs.

The ARI and VI showed that the clustering solutions with the same number of FC states detected by each of the clustering algorithms were dissimilar. Interestingly, for each *K*, with *K*∈{2, …, 20}, the FC states (centroids/medoids) detected by each of the clustering algorithms with significant intergroup differences in the mean fractional occupancy and mean dwell time (*p* < α_2_, two-tailed tests) were found to be highly correlated. Therefore, both the *K*-means and the *K*-medoids algorithms were found to effectively identify similar FC states whose properties provide the capacity to differentiate SZ patients from HCs.

## 4. Discussion

This study investigated differences in resting-state brain activity between schizophrenia patients and healthy controls. This was done from the perspective of dynamical systems theory by considering the exploration of functional networks as trajectories through a state space—providing an insightful framework to interpret brain activity alterations in schizophrenia.

Overall, SZ patients were found to spend less time in a globally synchronized state, or Global Mode, in line with previous studies using different analytical approaches (Damaraju et al., 2014; Rabany et al., 2019; Sanfratello et al., 2019). Conversely, a repertoire of non-global FC states, involving the phase synchronization of brain areas belonging to the Somatomotor, Dorsal Attention and Limbic RSNs, were shown to recur more often in SZ patients. These non-global FC states have been previously referred to as “ghost” attractor states since they appear briefly and erratically, yet consistently and recurrently across subjects (Vohryzek et al., 2020). The detection of increased excursions to a subset of these “ghost” attractor states in schizophrenia is suggestive of alterations in the energy landscape of brain activity. In particular, the RSNs that recurred more often have been previously associated with schizophrenia symptoms. In fact, the Somatomotor network was related to motor and negative symptoms of schizophrenia (Bernard et al., 2017), the Limbic network was related to positive symptoms (such as paranoid ideation; Walther et al., 2021) and to disorganization (Lin et al., 2018) and the Dorsal Attention network was related to the regulation of attention Kandilarova et al. (2021), as well as to positive and negative symptoms' improvement after antipsychotic treatment (Kraguljac et al., 2016).

Regarding state-to-state transitions, SZ patients were found to be less likely to remain in the globally synchronized state, in line with previous studies (Rabany et al., 2019). Importantly, this state has been linked to greater neural flexible switching *via* integration or segregation of different functional connections (Cabral et al., 2017; Nomi et al., 2017). Therefore, the reduced ability of SZ patients to access and remain in this state could be hypothesized to provoke impaired integration of functionally meaningful networks (Dong et al., 2018). Additionally, SZ patients were found to have a higher probability of transitioning from the Global Mode to states related to the Somatomotor and Limbic RSNs and from a Dorsal Attention-related network to a Limbic-related network—reinforcing the role of brain activity alterations across these “ghost” attractor states in schizophrenia. Another interesting finding was the reduced ability to remain in a state related to the Default RSN in SZ patients. In fact, this RSN has been linked to core processes of human cognition (Greicius et al., 2003; van den Heuvel and Hulshoff Pol, 2010)—support the view of schizophrenia as a disorder affecting cognitive function. Finally, the reduced ability of SZ patients to remain in an Orbitofrontal Network—a network hypothesized to be involved in sensory integration, monitoring the reward value of reinforcers, decision making and expectation (Kringelbach and Rolls, 2004). This could potentially explain some of the positive and negative symptoms associated with this disorder. These findings provide additional evidence of the altered energy landscape in schizophrenia. However, how these alterations translate into distorted cognition and behavior remains completely unclear and future investigations should gather a diverse panel of experts to explore how these findings could be applied to improve our understanding of schizophrenia.

Despite the lack of a full understanding of the relationship between connectivity patterns observed in Electroencephalography (EEG) and fMRI, these findings could be speculated to portray a temporal dynamics related to that observed with EEG microstates measured at a different time resolution. In fact, in line with the aforementioned findings, EEG studies have reported an increased occurrence of a microstate associated with the Limbic RSN (Ramos da Cruz et al., 2020) and unexpectedly more transitions from a microstate associated with the Attention RSN to a microstate associated with the Limbic RSN in schizophrenia (Rieger et al., 2016).

On the question of the influence of using the *K*-medoids algorithm to conduct an LEiDA analysis, this study found that similar intergroup differences are captured by employing either the *K*-medoids algorithm or the *K*-means algorithm. This finding suggests that the choice of an optimal clustering algorithm should rely not only on statistical and cluster validation analyses, but also on concepts and methods from dynamical systems theory (Deco and Jirsa, 2012; Cabral et al., 2017; Vohryzek et al., 2020). On the one hand, from the definition of the *K*-means algorithm, the detected FC states (centroids) are not necessarily observations from the input dataset, but could rather be interpreted as averaged recurrent unobserved FC patterns; hence their designation as “ghost” attractor states (Vohryzek et al., 2020). However, the definition of the *K*-medoids algorithm implied the detected FC states (medoids) are observed recurrent FC patterns. Research questions pertaining to the functional meaning of the detected FC patterns underline the need to employ tools from dynamical systems theory to provide further insights into the dynamical regime of brain activity (Deco and Jirsa, 2012; Cabral et al., 2017; Vohryzek et al., 2020).

Developing on from previous LEiDA analyses, this study proposes examining the limiting probability of FC states. This property offers valuable insights into the long-run proportion of time that a DTMC spends in each state. Specifically, this measure is computed from the TPMs which characterize the state trajectories—capturing dynamic behavior of brain activity to a greater extent than fractional occupancy. However, considerably more research will need to be conducted to determine its utility. Furthermore, the measurements of this property are derived from the estimation of the stationary distribution of the state trajectories, defined as irreducible and aperiodic DTMCs. A natural progression of this work is to examine whether intergroup differences in the stationary distributions provide further insights into the limiting dynamic behavior of brain activity in diseased and healthy populations. This could be achieved by employing the two-sample goodness of fit χ^2^ test.

One shortcoming of this study which could have affected the measurements of both the state-to-state transition and state limiting probabilities is the low temporal resolution of the neuroimaging data (TR = 2 s). This was most clearly observed from the inconsistencies found across state trajectories obtained from the optimal clustering solution where, oftentimes, the occurrence of all FC states was not guaranteed. In fact, Magnetoencephalography (MEG) studies have suggested that brain functional connectivity dynamics occurs at time scales of approximately 200 ms (Baker et al., 2014; Vidaurre et al., 2016). Accordingly, future work should utilize data with higher temporal resolution to enable the capture of more rapid dynamics—improving the utility of these properties as possible biomarkers of schizophrenia.

Another limitation of this study is that the detected FC states were strongly constrained by the selected parcellation atlas (AAL). Despite having shown consistent results across studies employing LEiDA (Cabral et al., 2017; Figueroa et al., 2019; Lord et al., 2019; Larabi et al., 2020; Vohryzek et al., 2020), the AAL template is based on an anatomical parcellation and, therefore, may not provide an adequate framework to conduct an analysis of dFC. Accordingly, future studies could extend this analysis to other fMRI-derived anatomical or functional parcellations.

The effect of variables such as age, gender and clinical history of patients were not taken into account while assessing intergroup differences. Specifically, intergroup differences were attributed exclusively to the effect of the group. Further research is required to determine whether these variables or their interaction could explain the variability found between groups. Another unaddressed issue was whether not applying nuisance regression strategies influenced the LEiDA method and therefore, the observed intergroup differences. Future investigations on this question could contribute with valuable insights into this controversial preprocessing step. Despite these weaknesses and supported by the complementary analysis presented in Supplementary Material Section 2, based on a large sample, this study provided unbiased and statistically rigorous evidence for differences between patients with schizophrenia and healthy controls—leading to increased confidence in the biomarking value of the findings from this research.

## 5. Conclusion

Resting-state dynamic functional connectivity comparisons were conducted between schizophrenia patients and healthy controls by employing and extending the LEiDA method. Through the characterization of the temporal expression of different FC states, this study found that schizophrenia patients exhibit an altered energy landscape of brain activity. An implication of this is the possibility that, even in the absence of any explicit task, schizophrenia patients transition more frequently to network patterns that are commonly activated during specific tasks.

## Data Availability Statement

The imaging data was collected and shared by the Mind Research Network and the University of New Mexico funded by a National Institute of Health Center of Biomedical Research Excellence (COBRE) grant 1P20RR021938-01A2. The datasets analyzed for this study can be found in the following repository: https://doi.org/10.6084/m9.figshare.4197885.v1.

## Ethics Statement

The studies involving human participants were reviewed and approved by National Ethics Committee. The patients/participants provided their written informed consent to participate in this study.

## Author Contributions

MF carried out the analysis and wrote the main manuscript. JC contributed with codes. JC and CA verified and advised the theoretical methods and supervised the whole project. PM was responsible for funding acquisition. MF, JC, and PM contributed in the final writing of the manuscript. All authors participated in the discussion of the ideas, read, and approved the submitted version.

## Funding

This work was funded by FLAD Science Award Mental Health 2021. This work was partially funded by National funds, through the Portuguese Foundation for Science and Technology (FCT)—projects UIDB/50026/2020 and UIDP/50026/2020. JC is funded by FCT grant CEECIND/03325/2017.

## Conflict of Interest

PM has received in the past 3 years grants, CME-related honoraria, or consulting fees from Angelini, AstraZeneca, Bial, Biogen, DGS-Portugal, FCT, FLAD, Janssen-Cilag, Gulbenkian Foundation, Lundbeck, Springer Healthcare, Tecnimede, Viatris, and 2CA-Braga outside of this study. The remaining authors declare that the research was conducted in the absence of any commercial or financial relationships that could be construed as a potential conflict of interest.

## Publisher's Note

All claims expressed in this article are solely those of the authors and do not necessarily represent those of their affiliated organizations, or those of the publisher, the editors and the reviewers. Any product that may be evaluated in this article, or claim that may be made by its manufacturer, is not guaranteed or endorsed by the publisher.
