# Supplementary material for: Increased Excursions to Functional Networks in Schizophrenia in the Absence of Task
Source: Front Neurosci. 2022 Mar 11;16:821179. doi: 10.3389/fnins.2022.821179 (PMC8963765; doi:10.3389/fnins.2022.821179)
Supplement: Supplementary file 1 [file Data_Sheet_1.pdf]

Supplementary Material

1 SUPPLEMENTARY TABLES AND FIGURES

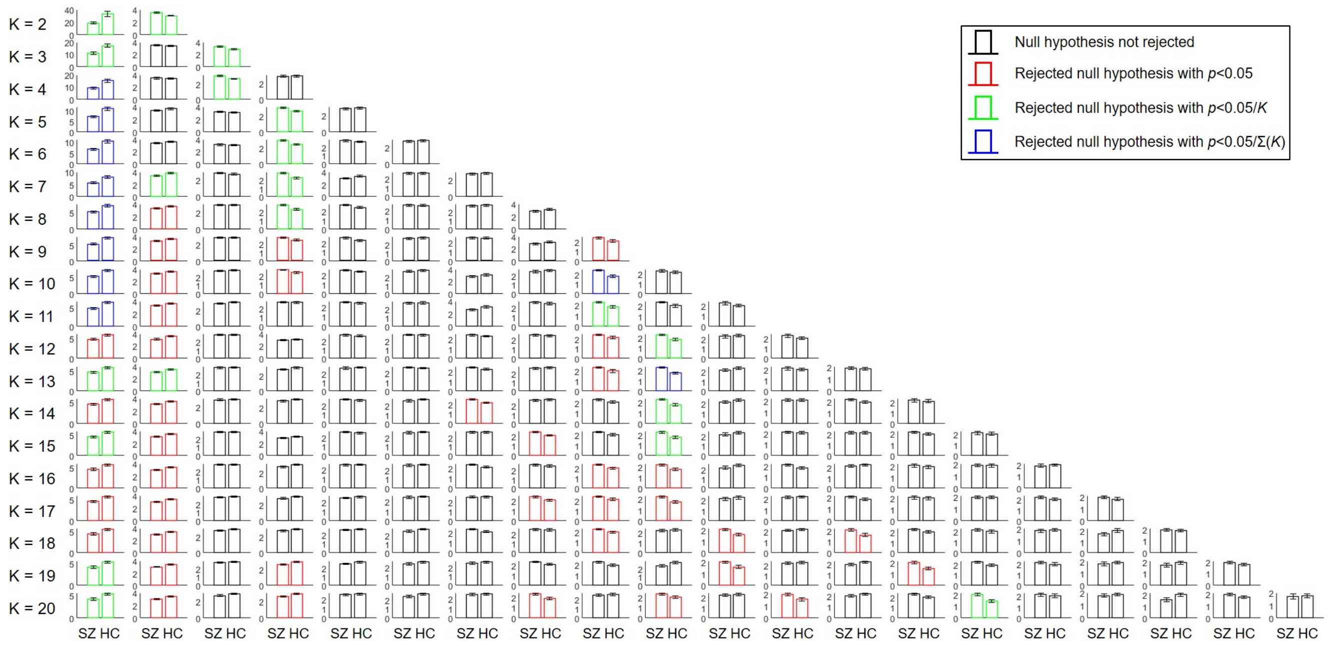

Figure S1. Intergroup comparisons of the mean dwell time of each FC state for each clustering solution. Barplot of the estimated mean dwell time with associated standard error of each FC state detected by the K-means algorithm for each group. For each FC state, the color of the bars indicates whether the null hypothesis of no intergroup differences in the mean dwell time was rejected (two-tailed tests). Black bars indicate the null hypothesis was not rejected at a 5% significance level. Red, green and blue bars indicate the null hypothesis was rejected at a 0.05, $0.05/K$ and $0.05/\sum_{K=2}^{20} K$ significance threshold, respectively. The standard error of each bar was calculated as the standard deviation of the sample data divided by the square root of the sample size.

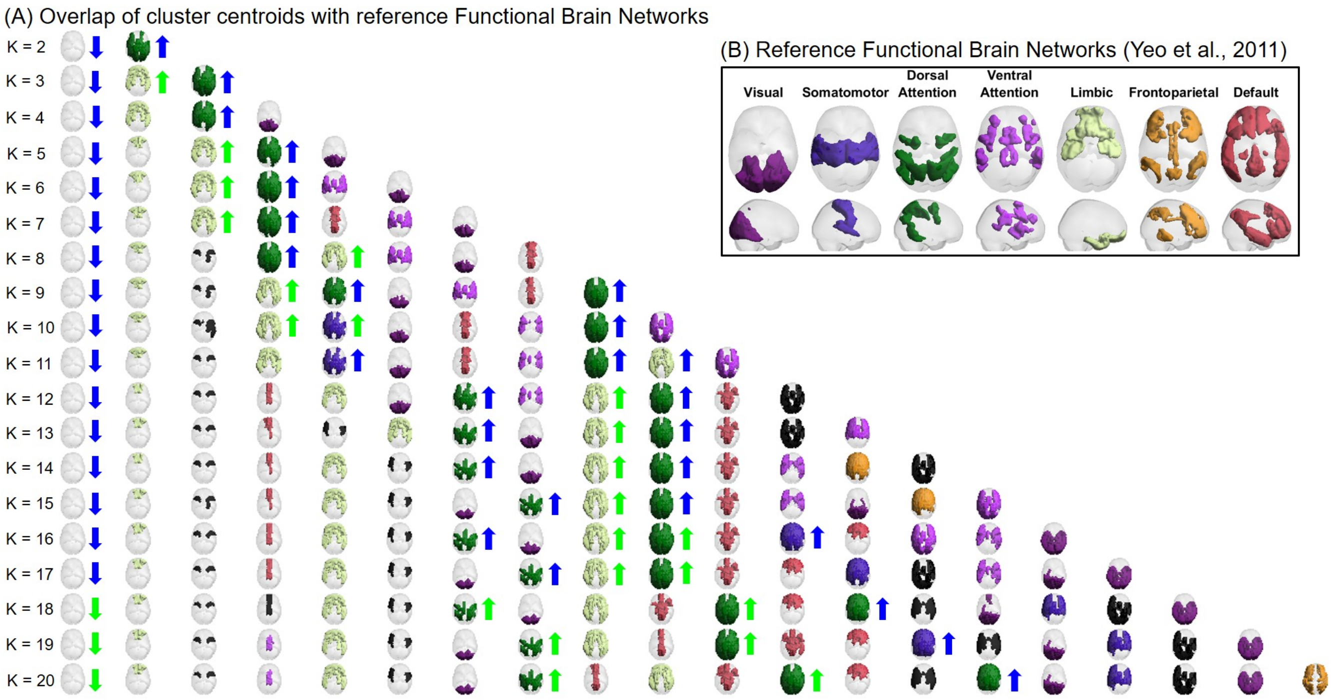

Figure S2. Overlap between FC states and Functional Brain Networks. **(A)** Representation of the centroids obtained for each clustering solution in cortical space. The rendered brain areas correspond to positive elements in the vectors of the centroids. Brain regions are colored according to the reference RSNs defined by Thomas Yeo et al. (2011) whose p-value obtained from computing the Pearson correlation coefficient was lowest (with $p < 0.05/K$). Centroids not significantly overlapping with any of the reference RSNs are colored in black. For each FC state, upward/downward arrows indicate the mean fractional occupancy was significantly increased/decreased in the schizophrenia group compared to the control group. Green and blue arrows indicate $p < \alpha_2$ and $p < \alpha_3$ (one-tailed tests), respectively. **(B)** Reference functional brain networks estimated by Thomas Yeo et al. (2011).

Table S1. Stability analysis of the optimal clustering solution. Results obtained across the 10 cross-validation folds for each clustering agreement measure.

	**Cross-validation fold number**									
**Indices**	1	2	3	4	5	6	7	8	9	10
**Percent agreement**	0.642	0.536	0.583	0.688	0.529	0.564	0.602	0.698	0.547	0.523
**Adjusted Rand**	0.692	0.712	0.717	0.757	0.696	0.678	0.749	0.718	0.773	0.709
**Variation of information**	1.379	1.504	1.349	1.237	1.396	1.402	1.291	1.229	1.181	1.388

2 INVESTIGATION OF THE IMPACT OF MOTION-RELATED SIGNAL ON THE GROUP RESULTS

In this study, the impact of residual motion-related signal on the group results derived using LEiDA was investigated following a lenient regime, designed to emulate a “worst” case scenario, according to which participants with high levels of gross motion, i.e., with a mean FD greater than 0.55 mm, were not considered for analysis (Parkes et al., 2018; Satterthwaite et al., 2012). This resulted in a sample with a total

of 118 participants - 52 SZ patients and 66 HCs - which was used to rerun the LEiDA analysis described in the main text. The state trajectories and FC states were derived using the K-means clustering algorithm.

Figure S3A presents the overlap between the detected FC states and the reference functional brain networks defined by Thomas Yeo et al. (2011), depicted in Figure S3B. Furthermore, Figure S3A shows the results obtained from evaluating whether the group mean fractional occupancy of a FC state differed between SZ patients and HCs.

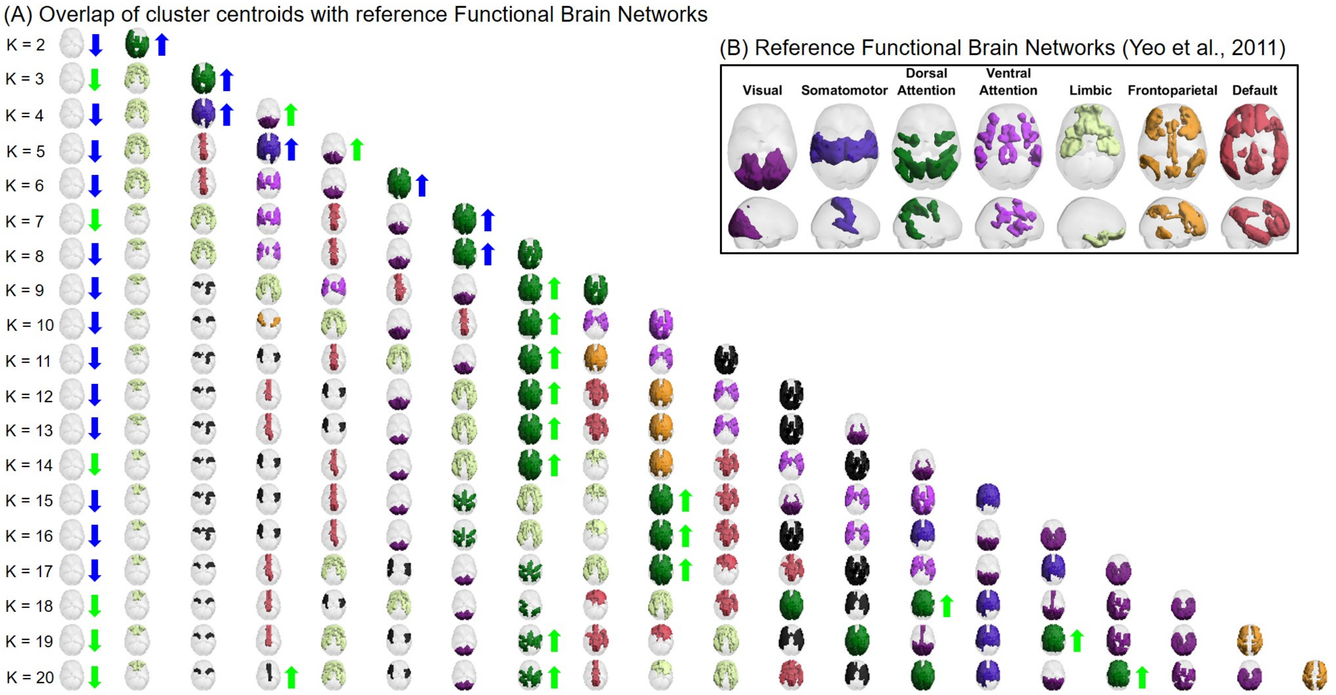

Figure S3. Overlap between FC states detected from the sample of 118 high-motion free participants and Functional Brain Networks. **(A)** Representation of the centroids obtained for each clustering solution in cortical space. The rendered brain areas correspond to positive elements in the vectors of the centroids. Brain regions are colored according to the reference RSNs defined by Thomas Yeo et al. (2011) whose p-value obtained from computing the Pearson correlation coefficient was lowest (with $p < 0.05/K$). Centroids not significantly overlapping with any of the reference RSNs are colored in black. For each FC state, upward/downward arrows indicate the mean fractional occupancy was significantly increased/decreased in the schizophrenia group compared to the control group. Green and blue arrows indicate $p < \alpha_2$ and $p < \alpha_3$ (one-tailed tests), respectively. **(B)** Reference functional brain networks estimated by Thomas Yeo et al. (2011).

Figure S3A shows that LEiDA detected a globally synchronized state (Global Mode) and a number of task-positive networks. Notably, similarly to the analysis considering all participants, the mean fractional occupancy of the Global Mode was significantly reduced in SZ patients compared to HCs across all clustering solutions ($p < \alpha_2$, one-tailed tests). Additionally, across clustering solutions, the mean fractional occupancy of FC states significantly overlapping with the Somatomotor, Dorsal Attention and Visual reference RSNs was significantly increased in SZ patients compared to HCs ($p < \alpha_2$, one-tailed tests). Interestingly, the mean fractional occupancy of FC states related to the Limbic canonical RSN did not differ between groups. Importantly, considering only the FC states for which significant differences in the mean fractional occupancy were detected, it was verified that the cluster centroids obtained from performing a LEiDA analysis on the complete sample of 145 participants and the cluster centroids derived from

applying LEiDA to the high-motion free sample of 118 participants were highly correlated - indicating that intergroup differences were detected in similar functional networks.

Figure S4A shows the results obtained from evaluating whether the group mean dwell time of a FC state differed between SZ patients and HCs. Similarly to the results presented in the main text, the mean dwell time of the Global Mode was significantly reduced in SZ patients compared to HCs ($p < \alpha_2$, one-tailed tests) and the mean dwell time of FC states overlapping significantly with the Dorsal Attention RSN estimated by Thomas Yeo et al. (2011), depicted in Figure S4B, was significantly increased in the SZ group compared to the HC group ($p < \alpha_2$, one-tailed tests).

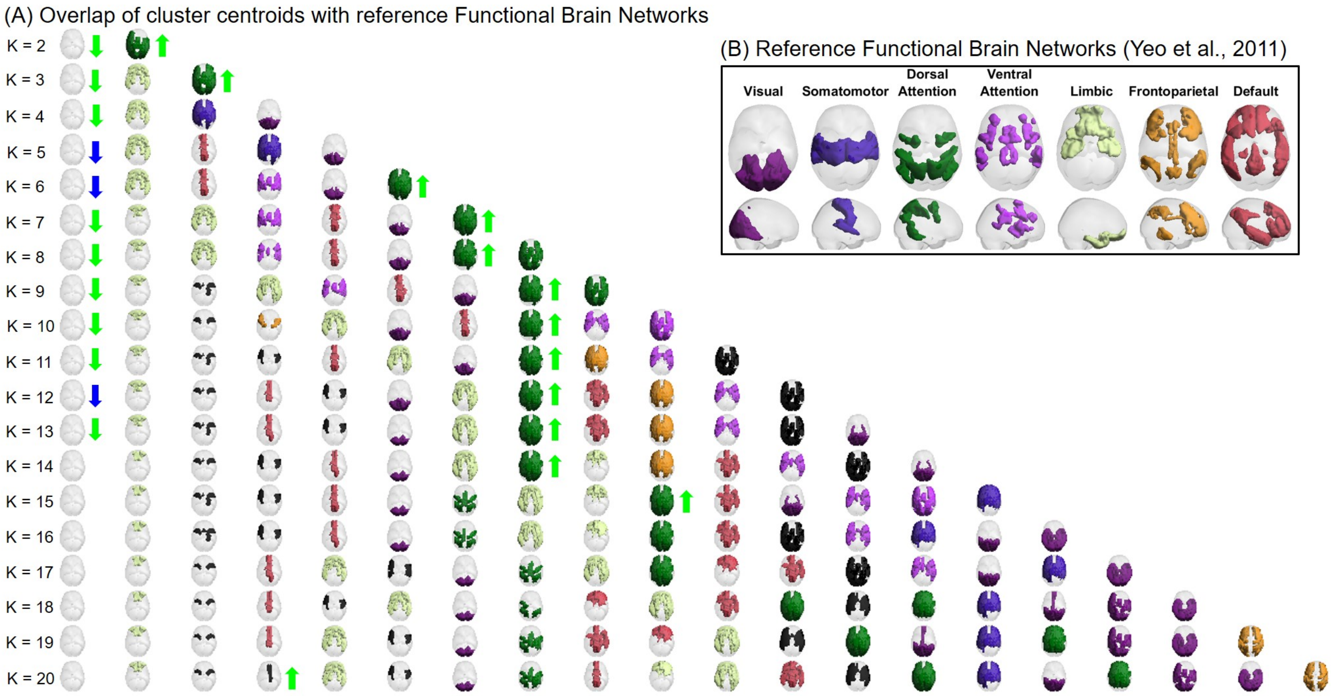

Figure S4. Overlap between FC states detected from the sample of 118 high-motion free participants and Functional Brain Networks. **(A)** Representation of the centroids obtained for each clustering solution in cortical space. The rendered brain areas correspond to positive elements in the vectors of the centroids. Brain regions are colored according to the reference RSNs defined by Thomas Yeo et al. (2011) whose p-value obtained from computing the Pearson correlation coefficient was lowest (with $p < 0.05/K$). Centroids not significantly overlapping with any of the reference RSNs are colored in black. For each FC state, upward/downward arrows indicate the mean dwell time was significantly increased/decreased in the schizophrenia group compared to the control group. Green and blue arrows indicate $p < \alpha_2$ and $p < \alpha_3$ (one-tailed tests), respectively. **(B)** Reference functional brain networks estimated by Thomas Yeo et al. (2011).

It must be noted that the previous analysis only excluded participants with high levels of gross motion. As such, following the previous analysis, under a stringent regime, participants were excluded if any of the following criteria were true: (1) mean FD greater than 0.25 mm; (2) more than 20% of the FDs were above 0.2 mm; and (3) if any FDs were greater than 5 mm (Parkes et al., 2018; Satterthwaite et al., 2013). Under this stringent regime, only the data from 2 participants (both healthy controls) was suitable for analysis. This reduced sample size was not appropriate to perform a LEiDA analysis.

Finally, “scrubbing”, i.e., censoring complete volumes with excessive motion, has been applied to remove the influence of motion-related fluctuations from the estimates of FC (Power et al., 2012). However,

in the current work, this option was not considered since, after censoring, the large number of within-subject censored volumes could potentially interrupt the temporal autocorrelation structure of the data (Lydon-Staley et al., 2019).

REFERENCES

Lydon-Staley, D. M., Ciric, R., Satterthwaite, T. D., and Bassett, D. S. (2019). Evaluation of confound regression strategies for the mitigation of micromovement artifact in studies of dynamic resting-state functional connectivity and multilayer network modularity. *Network Neuroscience* 3, 427–454. doi:10.1162/netn_a_00071

Parkes, L., Fulcher, B., Yücel, M., and Fornito, A. (2018). An evaluation of the efficacy, reliability, and sensitivity of motion correction strategies for resting-state functional mri. *NeuroImage* 171, 415–436. doi:https://doi.org/10.1016/j.neuroimage.2017.12.073

Power, J. D., Barnes, K. A., Snyder, A. Z., Schlaggar, B. L., and Petersen, S. E. (2012). Spurious but systematic correlations in functional connectivity mri networks arise from subject motion. *NeuroImage* 59, 2142–2154. doi:https://doi.org/10.1016/j.neuroimage.2011.10.018

Satterthwaite, T. D., Elliott, M. A., Gerraty, R. T., Ruparel, K., Loughead, J., Calkins, M. E., et al. (2013). An improved framework for confound regression and filtering for control of motion artifact in the preprocessing of resting-state functional connectivity data. *NeuroImage* 64, 240–256. doi:https://doi.org/10.1016/j.neuroimage.2012.08.052

Satterthwaite, T. D., Wolf, D. H., Loughead, J., Ruparel, K., Elliott, M. A., Hakonarson, H., et al. (2012). Impact of in-scanner head motion on multiple measures of functional connectivity: Relevance for studies of neurodevelopment in youth. *NeuroImage* 60, 623–632. doi:https://doi.org/10.1016/j.neuroimage.2011.12.063

Thomas Yeo, B. T., Krienen, F. M., Sepulcre, J., Sabuncu, M. R., Lashkari, D., Hollinshead, M., et al. (2011). The organization of the human cerebral cortex estimated by intrinsic functional connectivity. *Journal of Neurophysiology* 106, 1125–1165. doi:10.1152/jn.00338.2011. PMID: 21653723
